# Comparative Effectiveness of Opioids and Opioid Substitutes on Pain and Health Related Quality of Life Among Cancer Survivors

**DOI:** 10.1002/cam4.71189

**Published:** 2025-09-08

**Authors:** Shirley K. Chan Sanchez, Samuel V. David, Efstathia Polychronopoulou, Mukaila Raji, Yong‐Fang Kuo

**Affiliations:** ^1^ John Sealy School of Medicine University of Texas Medical Branch Galveston Texas USA; ^2^ School of Public and Population Health University of Texas Medical Branch Galveston Texas USA; ^3^ Office of Biostatistics, Department of Biostatistics & Data Science University of Texas Medical Branch Galveston Texas USA; ^4^ Division of Geriatric Medicine, Department of Internal Medicine University of Texas Medical Branch Galveston Texas USA; ^5^ Department of Biostatistics & Data Science, School of Public and Population Health University of Texas Medical Branch Galveston Texas USA

**Keywords:** cancer survivorship, comorbidity burden, gabapentinoids, health‐related quality of life, opioid alternatives, opioids, pain interference, prescription patterns

## Abstract

**Introduction:**

Chronic pain is a major but modifiable contributor to poor quality of life among long‐term cancer survivors. With growing concern over opioid‐related risks, gabapentinoids have emerged as a safer alternative, though evidence comparing their effectiveness remains limited.

**Methods:**

We conducted a retrospective cohort study using SEER‐MHOS linked data (1998–2021) to examine pain interference and health‐related quality of life (HRQoL) among 24,651 cancer survivors. Participants were categorized as opioid‐only (OPIOID‐only), gabapentinoid‐only (GABA‐only), both (BOTH), or no medication (NONE). Changes in pain interference and Physical and Mental Component Summary scores (PCS, MCS) were analyzed using paired t‐tests and multivariate regression, adjusting for demographic and clinical covariates.

**Results:**

Over a 1 to 3 year follow‐up, all groups showed increased pain interference and declines in PCS and MCS scores. PCS declined by −1.01 in the OPIOID‐only group and −1.06 in the GABA‐only group. The BOTH group had stable PCS (+0.06) and a modest improvement in pain interference (−0.07). Multivariate models showed no significant difference between OPIOID‐only and GABA‐only for pain interference or PCS, but the BOTH group had significantly less pain worsening (*p* = 0.0001) and PCS decline (*p* = 0.004).

**Conclusion:**

Gabapentinoids demonstrate comparable effectiveness as opioids for pain and HRQoL in cancer survivors, supporting their use as a safer alternative. Combination therapy showed better physical function and pain control, but findings may reflect higher baseline comorbidity and limited decline capacity rather than true superiority. These results underscored the need for personalized, multimodal pain management and further research on the long‐term safety of combination therapy.

## 1 Introduction

The cancer survivor population continues to grow in the United States, with approximately 18.1 million cancer survivors as of January 2022, a number projected to pass 26 million by 2040 [1]. This increase reflects improvement in early detection and more effective treatment, with the overall age‐adjusted cancer death rate declining by 33% from 1991 to 2021 [2]. While this represents significant progress in cancer care, survivors often face chronic health challenges, with pain being one of the most prevalent and impactful symptoms affecting their quality of life [3, 4]. Such challenges also pose psychosocial and financial burdens.

Prescription opioids remain central to pain management during active cancer treatment [5]. However, their long‐term use among cancer survivors has become increasingly concerning, particularly given the limited evidence supporting sustained effectiveness in chronic pain management and growing recognition of adverse outcomes [6, 7]. Recent studies have documented high rates of prolonged opioid therapy among cancer survivors, with rates varying significantly by cancer type and geographical region [8, 9]. Studies have also shown that5 to 100% of cancer survivors continue opioid use beyond 6 months post‐treatment, despite limited evidence supporting improved pain outcomes or functional status [10, 11]. Extended use leads to tolerance and physiological dependence, necessitating dose escalation patterns that significantly increase the risk of developing opioid use disorder (OUD), with recent data suggesting that approximately one in five cancer survivors on long‐term opioid therapy may be at risk for OUD [12]. Long‐term opioid therapy remains common among older cancer survivors, with variations based on cancer type and demographics, but studies suggest that extended opioid use does not necessarily translate to improved pain outcomes or quality of life [9].

The adverse health impacts of these prescribing patterns are reflected in healthcare utilization trends. Between 2006 and 2015, opioid‐related emergency department visits among cancer patients increased from 2078 to 5324, where the majority of these visits involved prescription opioids (94.3%), with heroin accounting for a smaller amount (5.7%) [13]. Additionally, studies indicate that cancer survivors experience significantly higher opioid prescribing rates (adjusted relative rate, 1.22; 95% CI: 1.11–134) compared to their non‐cancer counterparts, with rates remaining elevated even a decade after diagnosis [14]. These trends may suggest a complex relationship between survivorship care and pain management strategies.

Following the 2016 CDC guidelines for chronic pain management and subsequent policy initiatives, overall opioid prescribing patterns have shown declining trends [15]. Such policies include the SUPPORT Act of 2018, which expanded access to addiction treatment and strengthened prescription drug monitoring programs, and the DEA's 2014 rescheduling of hydrocodone combination products to Schedule II [16, 17]. However, recent evidence suggests these policies may have limited effectiveness in addressing pain management needs of cancer survivors. Concurrent with decreasing opioid prescriptions, alternative pain management approaches, particularly gabapentinoids, have seen increased utilization, rising by over 64% between 2012 and 2019 [18, 19]. Despite the shift in prescribing patterns, evidence regarding the comparative effectiveness of these alternatives in managing cancer‐related chronic pain remains limited, particularly concerning long‐term outcomes [6, 7].

To address these knowledge gaps, we examined trends in opioid and opioid‐alternative medication use, specifically gabapentinoid, among cancer survivors using the Surveillance, Epidemiology and End Results (SEER) cancer registry linked to Medicare Health Outcomes Survey (MHOS) data from 1998 to 2021. Additionally, we compared pain scores and health‐related quality of life measures across survivors using different pain management approaches including opioids, gabapentinoids, and combination (opioid + gabapentinoids) therapy.

## 2 Methods

### 2.1 Data Source

This study is a retrospective cohort analysis using the Surveillance, Epidemiology, and End Results—Medicare Health Outcomes Survey (SEER‐MHOS) data resources. The SEER provides accurate and reliable patient data related to demographics, initial treatment, tumor characteristics, and cause of death. The SEER program began in 1973 and covers about 45.9% of the US population, including 20 registries [20]. The MHOS, which is conducted annually, started in 1998 as a longitudinal survey conducted by the Centers for Medicare and Medicaid Services to gather patient‐reported clinical data on health‐related quality of life (HRQoL), functional status, and comorbid conditions among a random sample of Medicare Advantage enrollees from each participating Medicare Advantage Organization (MAO) [21]. Sampling criteria, including eligibility requirements and participating MAOs, have changed over time during the study period. Therefore, the SEER‐MHOS linked data source provides an opportunity to examine patient‐reported outcomes among Medicare Advantage beneficiaries with cancer diagnoses. The Institutional Review Board of the University of Texas Medical Branch approved the study.

### 2.2 Cohort Selection

The study population includes 287,600 cancer survivors with any of the cancers of breast, lung, colorectal, prostate, uterus, skin, kidney, bladder, pancreas, or ovary, diagnosed at least 5 years before the baseline MHOS survey. Further inclusion criteria required continuous Medicare Part D enrollment for at least 12 months from their first survey date or until death to capture complete medication use data. Since Medicare Part D data became available starting in 2006, we restricted our analyses to participants whose first survey occurred in 2006 or later to ensure coverage. We only included participants who had a first follow‐up survey conducted between 1 and 3 years after the baseline survey (Table 2a). A total of 24,651 participants met these criteria and were included in the final cohort.

### 2.3 Drug Measurements

We evaluated opioid and gabapentinoid medication usage using their respective National Drug Codes (NDCs) from Medicare Part D data. Specifically, we extracted all opioid and gabapentinoid‐related records during the one‐year period following the baseline survey. From these records, we identified any opioid and/or gabapentinoid prescriptions filled within the one‐year period following the baseline survey (Table 2a). The presence of these medications during the window was used to determine index medication exposure and categorized participants accordingly. Based on the final medication records, study participants were classified into one of the four mutually exclusive categories of (1) Opioid‐only users (OPIOID‐only) (*n* = 5444; 22.1%), (2) Gabapentinoid‐only users (GABA‐only) (*n* = 1043; 4.2%) (3) Opioids + Gabapentinoid users (BOTH) (*n* = 1405; 5.7%), and (4) No opioid or gabapentinoid users (NONE) (*n* = 16,759; 67.9%).

### 2.4 Outcomes and Measures

The primary outcomes assessed pain interference and health‐related quality of life (HRQoL), measured through the MHOS surveys. Pain interference was assessed using self‐reported responses, which includes validated questions on how pain affects daily activities and physical functioning. Specifically, participants were asked: “During the past four weeks, how much did pain interfere with your normal work (including work outside the home and housework)?” Responses were recorded on a five‐point scale, with options ranging from “Not at all,” “A little bit,” “Moderately,” “Quite a bit,” to “Extremely.” Changes in pain interference were determined by comparing baseline and follow‐up responses; positive changes indicated worsening pain interference and negative changes reflected improvement over time. Use of this single pain‐interference item as an independent longitudinal outcome has been supported in prior cohort studies and psychometric work [22, 23, 24].

HRQoL was assessed using norm‐based the Physical Component Summary (PCS) and Mental Component Summary (MCS) *T* scores (mean = 50, SD = 10) derived from the SF‐36 or, in later MHOS waves, the VR‐12 health surveys. For VR‐12 respondents, SEER‐MHOS provides PCS/MCS values that have already been cross‐walked to the SF‐36 norm‐based metric using the published algorithm [25, 26, 27, 28]. Higher scores indicate better physical and mental health. The PCS score reflects physical health status, capturing limitations in physical activities, bodily pain, general health perceptions, and physical functioning. It is derived from responses assessing a patient's ability to perform activities such as climbing stairs, walking various distances, and performing self‐care tasks. Higher scores indicate greater physical functioning and less impairment due to pain or other health conditions, while lower scores suggest more severe physical limitations.

The MCS score evaluates mental health and emotional well‐being, incorporating areas such as social functioning, emotional role limitations, psychological distress, and energy levels. Questions in this section measure mood‐related symptoms, including anxiety, depression, and feelings of vitality, with higher scores representing better mental well‐being, whereas lower scores indicate greater distress, social impairment, or mood disturbances. Changes in PCS and MCS were determined by comparing baseline and follow‐up responses; a positive score indicated better PCS and MCS, and a negative score reflected worsening over time.

### 2.5 Covariates

Age at baseline survey was calculated from the duration between the year of birth (administrative files) to the year of baseline survey. Sociodemographic factors were obtained from both self‐reported and administrative sources. Race/ethnicity, education, and smoking status were obtained from the participants self‐reported information in the MHOS survey. Marital status and geographic residency were obtained from the enrollment database maintained by the Centers for Medicare & Medicaid Services. Cancer‐related factors (diagnosis year, disease stage and SEER regions) were obtained from the SEER‐Patient Entitlement and Diagnosis Summary File (PEDSF). The part D enrollment, original entitlement, and dual status were obtained from the CMS member files.

Self‐reported comorbid conditions were obtained from the Medicare Health Outcomes Survey (MHOS) itself. The CDC Chronic Disease Classification was used as a reference to guide the selection of conditions for inclusion in this study [29]. We included hypertension, congestive heart failure, coronary artery disease, stroke, arthritis, chronic lung disease (COPD), depression, diabetes, and osteoporosis, which were 9 out of the 19 from the CDC (excluding cancers) Classification [29]. Furthermore, we categorized the comorbidity burden as having 0, 1, 2, or 3+ comorbid conditions.

### 2.6 Statistical Analysis

We used bivariate analysis with chi‐squared statistics to describe the distribution of characteristics within the 4 medication usage groups. Baseline and follow‐up values for pain interference, Physical Component Summary (PCS), and Mental Component Summary (MCS) scores were compared within subjects using paired t‐tests to assess changes over time. To further evaluate whether the magnitude of these changes varied by medication usage group, we constructed unadjusted and adjusted linear regression models for each of these outcomes. We conducted a sensitivity analysis excluding those diagnosed before the year 2000 for these outcomes. All statistical tests were two‐sided with an alpha level of 0.05. Analyses were performed using SAS version 9.4.

## 3 Results

### 3.1 Characteristics of Study Participants and Baseline Pain Interference and HRQoL

Demographic and clinical characteristics differed across the four medication groups, which highlight variability in age, diagnosis year, and geographic distribution (Table 1). This may reflect shifting prescribing patterns, patient health profiles, and regional treatment preferences over time. Age at baseline survey varied by group; the BOTH group had the highest proportion of younger patients (7.1%) aged23 to 599 years, while the OPIOID‐only group had 2.9% in this range. At the time of the survey administration, 95.7% of participants were aged 65 years and older. In addition, patients in the GABA‐only group were more commonly diagnosed within the period 2005 to 2014 (47.9%), followed by 41.5% in the BOTH group, suggesting increased use of gabapentinoids in more recent years. Regional differences were observed as well, with a higher proportion of patients residing in the Northeast in the GABA‐only group (31.5%) compared to the OPIOID‐only group (23.7%), and a higher proportion residing in the South in the BOTH group (28.5%) compared to the OPIOID‐only group (24.5%).

**TABLE 1:** Baseline demographic, clinical, and cancer‐related characteristics.

Characteristics	Usage group				*p*‐value
	Opioid only (*N* = 5444)	Gaba only (*N* = 1043)	Both (*N* = 1405)	None (*N* = 16,759)	
Age at survey (years)					< 0.0001
23–59	161 (2.9)	29 (2.7)	100 (7.1)	215 (1.3)	
60–64	199 (3.7)	34 (3.3)	97 (6.9)	231 (1.4)	
65–70	1183 (21.7)	223 (21.4)	305 (21.7)	3857 (23.0)	
71–91	3901 (71.7)	757 (72.6)	903 (64.3)	12,456 (74.3)	
Race/ethnicity^a^					< 0.0001
White	4106 (75.4)	692 (66.4)	954 (67.9)	12,539 (74.8)	
Black	565 (10.4)	126 (12.1)	208 (14.8)	1419 (8.5)	
Hispanic	569 (10.5)	144 (13.8)	184 (13.1)	1526 (9.1)	
Asian/Pacific Islander/other	204 (3.75)	81 (7.8)	59 (4.2)	1275 (7.6)	
Gender					< 0.0001
Female	2925 (53.7)	649 (62.2)	894 (63.6)	8653 (51.6)	
Male	2519 (46.3)	394 (37.8)	511 (36.4)	8106 (48.4)	
Marital status					< 0.0001
Married	2435 (44.7)	370 (35.5)	561 (39.9)	7509 (44.8)	
Widowed	358 (6.6)	65 (6.2)	96 (6.8)	968 (5.8)	
Divorced/separated	439 (8.1)	74 (7.1)	139 (9.9)	1000 (6.0)	
Single	364 (6.7)	71 (6.8)	108 (7.7)	978 (5.8)	
Unknown	1848 (34.0)	463 (44.4)	501 (35.7)	6304 (37.6)	
Cancer site					< 0.0001
Breast	1774 (32.6)	391 (37.5)	537 (38.2)	5317 (31.7)	
Colorectal	664 (12.2)	139 (13.3)	167 (11.9)	2125 (12.7)	
Prostate	1610 (29.6)	234 (22.4)	318 (22.6)	5418 (32.3)	
Others	1396 (25.6)	279 (26.8)	383 (27.3)	3899 (23.3)	
Cancer stage					< 0.0001
In situ	615 (11.3)	133 (12.8)	167 (11.9)	1795 (10.7)	
Local	2356 (43.3)	489 (46.9)	654 (46.55)	7515 (44.8)	
Regional	654 (12.0)	151 (14.5)	226 (16.1)	1900 (11.34)	
Distant	80 (1.5)	16 (1.5)	23 (1.6)	172 (1.0)	
Unknown	1739 (31.9)	254 (24.4)	335 (23.8)	5377 (32.1)	
Cancer status					0.4923
Single	4982 (91.5)	948 (90.9)	1268 (90.3)	15,295 (91.3)	
Multiple	462 (8.5)	95 (9.1)	137 (9.8)	1464 (8.7)	
Year of diagnosis					< 0.0001
1973–2000	1979 (36.4)	251 (24.1)	426 (30.3)	5936 (35.4)	
2001–2004	1706 (31.3)	292 (28.0)	396 (28.2)	5042 (30.1)	
2005–2014	1759 (32.3)	500 (47.9)	583 (41.5)	5781 (34.5)	
Comorbidity count^a^, ^d^					< 0.0001
0	461 (8.5)	74 (7.1)	62 (4.4)	2427 (14.5)	
1	1095 (20.1)	185 (17.7)	189 (13.5)	4984 (29.7)	
2	1253 (23.0)	254 (24.4)	299 (21.3)	4292 (25.6)	
3+	2635 (48.4)	530 (50.8)	855 (60.9)	5056 (30.2)	
Education^a^					< 0.0001
Less than high school	1066 (19.6)	242 (23.2)	325 (23.1)	2731 (16.3)	
High school/some college	3015 (55.4)	563 (54.0)	781 (55.6)	9176 (54.8)	
College graduate	1213 (22.3)	207 (19.9)	255 (18.1)	4494 (26.8)	
Unknown	150 (2.8)	31 (3.0)	44 (3.1)	358 (2.1)	
Poverty^c^					0.22
Lowest	1247 (22.9)	255 (24.4)	326 (23.2)	4022 (24.0)	
Low to moderate	1270 (23.3)	245 (23.5)	320 (22.8)	4096 (24.4)	
Moderate to high	1325 (24.3)	240 (23.0)	359 (25.5)	4009 (23.9)	
Highest	1369 (25.2)	251 (24.1)	345 (24.6)	3976 (23.7)	
Unknown	233 (4.3)	52 (5.0)	55 (3.9)	656 (3.9)	
Dual enrollment					< 0.0001
Yes	638 (11.7)	198 (20.0)	281 (20.0)	1388 (8.3)	
No	4806 (88.3)	845 (80.0)	1124 (80.0)	15,371 (91.7)	
Original entitlement					< 0.0001
Disability or ESRD	988 (18.2)	183 (17.6)	448 (31.9)	1431 (8.5)	
Age based eligibility	4456 (81.9)	860 (82.5)	957 (68.1)	15,328 (91.5)	
Smoking status^a^					< 0.0001
Yes	483 (8.9)	66 (6.3)	175 (12.5)	973 (5.8)	
No	4860 (89.3)	957 (91.8)	1196 (85.1)	15,516 (92.6)	
Unknown	101 (1.9)	20 (1.9)	34 (2.4)	270 (1.6)	
Geographic residency^b^					0.003
Big metro	2982 (54.8)	621 (59.5)	785 (55.9)	9280 (55.4)	
Metro	1982 (36.4)	333 (31.9)	470 (33.5)	6073 (36.2)	
Others	480 (8.8)	89 (8.5)	150 (10.7)	1406 (8.4)	
SEER region					< 0.0001
West	2379 (43.7)	380 (36.4)	600 (42.7)	6832 (40.8)	
Northeast	1291 (23.7)	329 (31.5)	297 (21.1)	5244 (31.3)	
Midwest	441 (8.1)	68 (6.5)	106 (7.5)	1409 (8.4)	
South	1333 (24.5)	266 (25.5)	402 (28.6)	3274 (19.5)	
Physical Component Summary score^a^ (Baseline) (Mean [SD])	36.5 (12.3)	34.9 (11.6)	30.1 (11.5)	42.2 (11.5)	< 0.0001
Mental Component Summary Score^a^ (Baseline) (Mean [SD])	51.4 (11.7)	51.1 (11.4)	47.6 (13.0)	53.9 (9.9)	< 0.0001
Pain interfering with work question^a^					< 0.0001
Not at all	1212 (22.3)	186 (17.8)	142 (10.1)	6798 (40.6)	
A little bit	1504 (27.6)	288 (27.6)	215 (15.3)	5243 (31.3)	
Moderately	1080 (19.8)	240 (23.0)	281 (19.9)	2626 (15.7)	
Quite a bit	1192 (21.9)	240 (23.0)	500 (35.6)	1601 (9.6)	
Extremely	401 (7.4)	69 (6.6)	249 (17.7)	342 (2.0)	
Missing	55 (1.0)	20 (1.9)	18 (1.3)	(149.9)	

^a^ Derived from the MHOS self‐reported data.

^b^ Based on rural–Urban Continuum Code for State/county of residence at time of survey.

^c^ Poverty was categorized based on total poverty rates at the county level using census tract data.

^d^ Comorbidities based on CDC Classification: Preventing Chronic Disease (available 9/19, including depression (has a doctor ever told you that you had depression) and arthritis (either of the hand/wrist or hip/knee) and excluding cancer).

The baseline pain interference level differed significantly in all medication groups compared to NONE group; Only 22.3% of patients in the OPIOID‐only group, 17.8% in the GABA‐only group, and 10.1% in the BOTH group reported “not at all” experiencing interference due to pain, compared to 40.6% in the NONE group (*p* < 0.0001). Conversely, the BOTH group had the highest proportion of patients reporting “quite a bit” or “extreme” pain interference (53.3%), whereas these proportions were 29.3% in the OPIOID‐only group, 29.6% in the GABA‐only group, and 11.6% in the NONE group. Baseline HRQoL also varied significantly by medication group. The NONE group demonstrated the highest mean PCS score at 42.2 (SD 11.5), followed by the OPIOID‐only group 36.5 (SD 12.3), GABA‐only group 34.9 (SD 11.6), and BOTH group 30.1 (SD 11.5). Similar patterns were observed for the MCS scores, with the NONE group having a mean of 53.9 (SD 9.9), followed by 51.4 (SD 11.7) in the OPIOID‐only group, 51.0 (SD 11.4) in the GABA‐only group, and 47.6 (SD 13.0) in the BOTH group.

### 3.2 Baseline Versus Follow‐Up Changes in Pain Interference and HRQoL

Between a 1–3 year follow‐up, paired t‐tests revealed all groups experienced increases in overall pain interference level and declines in HRQoL (Table 2). Baseline to follow up change for overall pain interference slightly increased in the OPIOID‐only group (+0.06, *p* < 0.0001) and in the NONE group (+0.07, *p* < 0.0001), while the BOTH group had a significant decrease (−0.07, *p* < 0.0.018). The GABA‐only group had an insignificant increase in pain interference level (+0.04, *p* = 0.216). With regard to PCS scores, significant declines were observed in the OPIOID‐only (−1.01, *p* < 0.0001), GABA‐only (−1.06, *p* = 0.0008), and NONE groups (−1.13, *p* < 0.0001). The BOTH group exhibited no change in PCS scores (+0.06, *p* = 0.806). MCS scores declined modestly in the OPIOID‐only group (−0.52, *p* = 0.0005), BOTH group (−0.63, *p* = 0.051), and NONE group (−0.46, *p* < 0.0001), whereas the GABA‐only group showed a non‐significant increase of +0.07 (*p* = 0.837).

**TABLE 2:** Baseline versus follow‐up change comparison.

(a) Baseline and follow‐up timeline overview 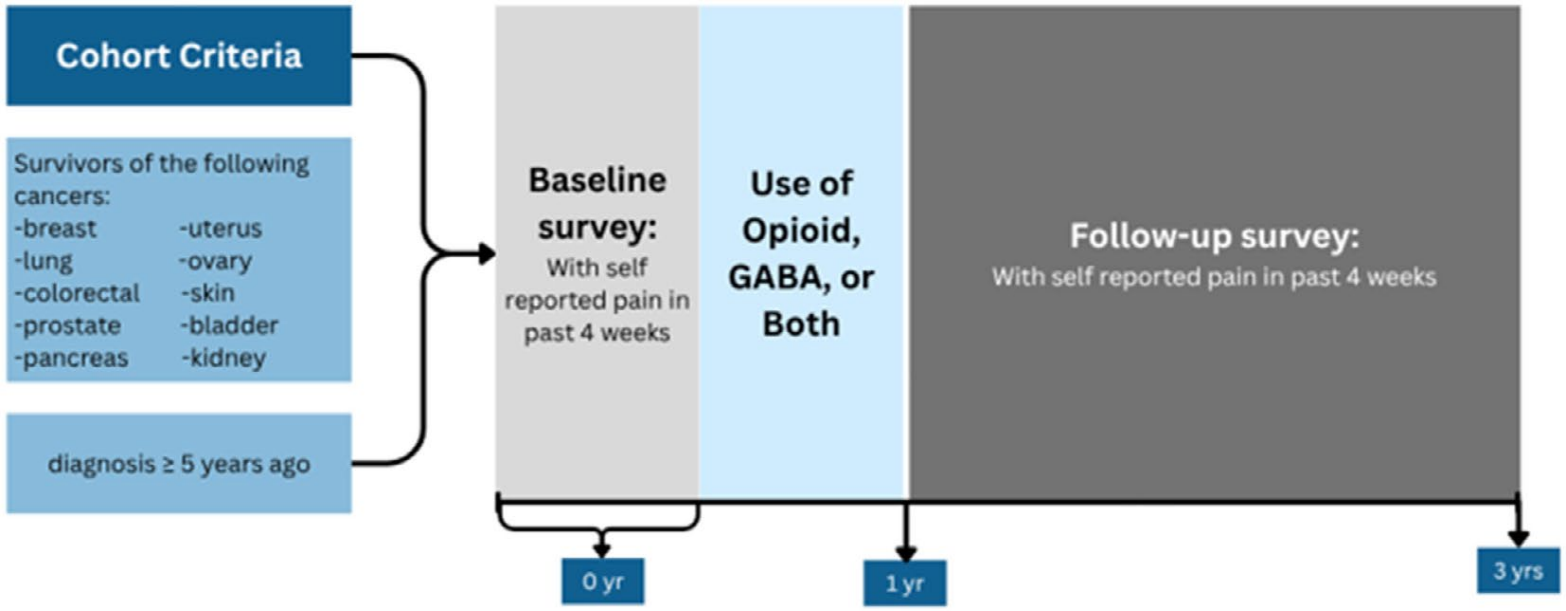

Outcome measure	Group									
	Opioid only		GABA only		Both		None		Total population	
	Mean change (SD)	*p*‐value^a^	Mean change (SD)	*p*‐value^a^	Mean change (SD)	*p*‐value^a^	Mean change (SD)	*p*‐value^a^	Mean change (SD)	*p*‐value^a^
(b) Baseline versus follow‐up change comparison										
Pain Inter ference	0.06 (1.14)	< 0.0001	0.04 (1.12)	0.2157	−0.07 (1.14)	0.019	0.07 (1.0)	< 0.0001	0.06 (1.04)	< 0.0001
PCS score	−1.01 (9.88)	< 0.0001	−1.06 (9.69)	0.0008	0.06 (9.55)	0.806	−1.13 (9.05)	< 0.0001	−1.03 (9.30)	< 0.0001
MCS score	−0.52 (10.46)	0.0005	0.07 (10.47)	0.837	−0.63 (11.35)	0.051	−0.46 (9.36)	< 0.0001	−0.46 (9.78)	< 0.0001

^a^ Paired *t*‐test.

### 3.3 Multivariate Analysis of Pain Interference and HRQoL

After adjusting for demographic and clinical covariates, multivariate analysis showed no significant differences in the increase of pain interference between the GABA‐only and OPIOID‐only groups (*p* = 0.602 for fully adjusted model, Table 3). Similarly, the GABA‐only and OPIOID‐only groups showed no significant differences in the decrease in PCS (*p* = 0.881 for fully adjusted model, Table 4). Multivariate analysis for change in MCS showed no differences in the GABA‐only group, BOTH group, and NONE group when compared to the OPIOID‐only group (*p* = 0.118, *p* = 0.400, *p* = 0.454 respectively, Table 5). The BOTH group had significantly less increased pain interference change (*p* = 0.0001 for the fully adjusted model, Table 3) and significantly less PCS decline (*p* = 0.004 for fully adjusted model, Table 4) compared to the OPIOID‐only group. We performed a sensitivity analysis by excluding patients diagnosed before 2000, for whom staging data were missing, and observed consistent results (Table S1). We also performed a stratified analysis for change in pain interference separately for baseline severity, selecting participants with pain levels1 to 33 (up to moderate pain) and4 to 55 (quite a bit and extreme pain) and we observed similar consistent results (Table S2).

**TABLE 3:** Multivariate analysis for change in pain interference.

Characteristics	Model 1 unadjusted		Model 2 adjusted for demographics		Model 3 adjusted for demographics and cancer characteristics	
	Estimate (95% CI)	*p*‐value	Estimate (95% CI)	*p*‐value	Estimate (95% CI)	*p*‐value
Group						
Opioid only	REF		REF		REF	
Gaba only	−0.02 (−0.09, 0.05)	0.581	−0.01 (−0.08, 0.05)	0.595	−0.01 (−0.08, 0.05)	0.602
Both	−0.13 (−0.19, −0.07)	< 0.0001	−0.133 (−0.196, −0.071)	< 0.0001	−0.12 (−0.18, −0.06)	0.0001
None	0.01 (−0.02, 0.04)	0.453	0.01 (−0.02, 0.04)	0.444	−0.006 (−0.038, 0.03)	0.736

*Note:* Demographics‐ age at survey, gender, race, year group, smoking category, area, SEER region, education. Cancer characteristics‐ cancer type, cancer status, comorbidities, stage.

**TABLE 4:** Multivariate analysis for change in PCS.

Characteristics	Model 1 unadjusted		Model 2 adjusted for demographics		Model 3 adjusted for demographics and cancer characteristics	
	Estimate (95% CI)	*p*‐value	Estimate (95% CI)	*p*‐value	Estimate (95% CI)	*p*‐value
Group						
Opioid only	REF		REF		REF	
Gaba only	−0.050 (−0.698, 0.598)	0.88	−0.031 (−0.68, 0.62)	0.922	−0.050 (−0.697, 0.598)	0.881
Both	1.077 (0.500, 1.654)	0.00003	0.976 (0.399, 1.553)	0.001	0.841 (0.264, 1.419)	0.004
None	−0.121 (−0.420, 0.177)	0.426	−0.076 (−0.376, 0.224)	0.618	0.148 (−0.156, 0.451)	0.339

*Note:* Demographics‐ age at survey, gender, race, year group, smoking category, area, SEER region, education. Cancer characteristics‐ cancer type, cancer status, comorbidities, stage.

**TABLE 5:** Multivariate analysis for change in MCS.

Characteristics	Model 1 unadjusted		Model 2 adjusted for demographics		Model 3 adjusted for demographics and cancer characteristics	
	Estimate (95% CI)	*p*‐value	Estimate (95% CI)	*p*‐value	Estimate (95% CI)	*p*‐value
Group						
Opioid only	REF		REF		REF	
Gaba only	0.590 (−0.091, 1.272)	0.090	0.573 (−0.110, 1.256)	0.100	0.545 (−0.138, 1.229)	0.118
Both	−0.108 (−0.715, 0.498)	0.726	−0.197 (−0.805, 0.411)	0.525	−0.262 (−0.870, 0.347)	0.400
None	0.058 (−0.256, 0.372)	0.719	0.069 (−0.247, 0.385)	0.670	0.122 (−0.198, 0.443)	0.454

*Note:* Demographics‐ age at survey, gender, race, year group, smoking category, area, SEER region, education. Cancer characteristics‐ cancer type, cancer status, comorbidities, stage.

## 4 Conclusion

### 4.1 Summary of Findings

This study examined the comparative effectiveness of opioids and gabapentinoids in managing pain and HRQoL among long‐term cancer survivors using the SEER‐MHOS dataset. Over the two‐year follow‐up period, all medication groups experienced worsening pain and declining physical and mental function over a 1–3 year follow‐up period. These findings emphasize that persistent pain remains a significant challenge in the survivorship phase, even among those receiving pain medications.

Gabapentinoid‐only users showed similar changes in pain interference and PCS scores compared to opioid‐only users, supporting their use as a potentially safer alternative to opioids. This is clinically relevant in the context of ongoing concerns about long‐term opioid use, including dependence, overdose, and limited evidence of functional benefit in chronic pain. Notably, participants in the BOTH group demonstrated the most favorable outcomes in pain interference and PCS scores, despite entering the study with the highest baseline pain levels and comorbidity burden. This is an unexpected finding—giving the increase in adverse drug events with co‐use of two CNS depressing drugs—opioids and GABA. It is not clear why the BOTH group had the most pain and PCS score benefit. This finding may reflect a true additive benefit of dual therapy in patients with complex needs. However, it is equally plausible that regression to the mean or floor effects among more debilitated individuals contributed to this observation. However, results from stratified analysis based on baseline pain level were similar. It is also possible that these patients may have had limited potential for further functional decline, making even small changes appear more favorable in comparison to less impaired groups. Future studies—especially qualitative interviewing of cancer survivors are—needed to examine change in function, pain, and quality of life in the context of co‐use of several analgesics, high pain burden, and multi‐morbidity.

### 4.2 Persistent Pain and Unmet Management Needs in Long‐Term Cancer Survivors

Despite advances in cancer treatment and pain management, chronic pain remains a significant burden for long‐term cancer survivors. In this study, all groups experienced declines in PCS and MCS scores and increases in pain interference over the 2‐year follow‐up period, highlighting the persistent nature of pain in this population. These findings align with a previous study indicating that cancer survivors face enduring health challenges; the study, using the SEER‐MHOS dataset, found that older adult survivors of certain cancers reported lower health‐related quality of life (HRQoL) compared to individuals without a cancer history, underscoring the long‐term impact of cancer and its treatment on survivors' well‐being [30]. The lack of significant differences in mental health outcomes across groups, reflected in the MCS scores, further highlights the inadequacy of medication‐only approaches to address the multifaceted burden of pain. These findings are consistent with prior studies indicating that cancer‐related pain often includes both physical and emotional components, which may stem from nerve injury, treatment toxicity, musculoskeletal strain, and psychological distress [30, 31]. For many survivors, pain is a chronic, evolving condition that requires more than symptom suppression.

This reinforces the need for a shift toward comprehensive, multimodal pain management that also emphasizes mental and physical function outcomes. Non‐pharmacologic interventions such as physical therapy, acupuncture, mindfulness‐based stress reduction, and cognitive‐behavioral therapy can address physical limitations and emotional suffering simultaneously [32]. Additionally, survivorship care planning should include routine screening for pain interference and mental health distress, especially in populations with high comorbidity burden.

### 4.3 Clinical Implications

As the opioid crisis continues to shape prescribing practices, identifying effective and lower risk alternatives has become a clinical priority. Gabapentinoids, originally developed for seizure disorders, are increasingly used to treat neuropathic and chronic musculoskeletal pain, particularly in cancer survivors. Their distinct mechanism of modulating calcium channels rather than mu‐opioid receptors makes them attractive as adjunctive or standalone therapies with a lower risk of euphoria, misuse, tolerance, and overdose [33, 34]. Moreover, their anxiolytic properties may offer added benefit in patients with comorbid mood disorders, a common feature among survivors experiencing chronic pain [35]. The increased use of gabapentinoids among more recently diagnosed patients and younger cancer survivors in this study, shown by the highest proportion of diagnoses between 2005 and 2014 in the GABA‐only group (47.9%) and the highest proportion of younger individuals aged 23 to 599 in the BOTH group (7.1%) illustrates evolving practice patterns and the growing acceptance of gabapentinoids as either primary or adjunctive pain management agents in oncology and primary care settings. In contrast, opioids act by binding to mu‐opioid receptors in the central nervous system, effectively blocking pain signals and inducing analgesia but may present various challenges including tolerance, dependence, opioid‐induced hyperalgesia, cognitive impairment, propensity for falls and fractures, and overdose—especially at higher doses when odds of respiratory depression increase [34, 36].

However, the combination of gabapentinoids and opioids must be approached with caution. Although the BOTH group had better physical function outcomes in this study, prior research in non‐cancer populations has shown mixed results. One study demonstrated that while combining these agents may offer superior analgesia in some settings, polypharmacy was also associated with worse pain control and no significant effect on anxiety [37]. Additionally, other studies have found that co‐prescribing opioids with gabapentinoids increases the risk of central nervous system depression and fall‐related injuries, raising safety concerns particularly for older or frail survivors [36, 38]. This study did not directly assess adverse drug events, and further research is warranted to evaluate the long‐term safety of combination therapy in survivorship care.

### 4.4 Study Limitations and Strengths

There are limitations that should be considered when interpreting these findings. First, this study relied on SEER‐MHOS self‐reported data, which may introduce recall bias and variability in how patients perceive and report pain. Second, the MHOS pain‐interference item has a 4‐week recall window, whereas medication exposure was identified over the 12‐month period after the baseline survey; hence, the time frames do not perfectly align and, also, we do not know how long before the survey they were taking these medications. Third, our study does not have claims data and only Medicare Part D, which provides medication prescriptions that were filled, not those that were actually taken by patients. The study did not look into the dosage and duration of opioid and gabapentinoid prescriptions due to limitations in Part D data. Also, GABA are primarily indicated for neuropathic pain; future research adding Medicare Part A and B data will help to identify the type of pain in studying treatment effectiveness [33]. Fourth, underlying pain indications were not directly accounted for, meaning that some groups may have had inherently different pain profiles. Also, this study does not include over‐the‐counter analgesics, which may contribute to pain management and could have influenced patient‐reported outcomes or masked the true extent of pain interference, especially among those in the NONE group. This highlights the need for further quantitative analysis—especially of survey data and—qualitative interviewing studies of survivors to fully understand the extent, type, and impact of non‐prescription medication use and non‐pharmacological approaches on pain and function outcomes. Finally, this study focuses on the Medicare advantage health population, limiting generalizability to younger cancer survivors who may have different pain management needs and medication responses. It is also important to note that because some cancer sites are sparsely represented, sub‐analyses by individual cancer type were under‐powered and therefore not performed.

This study uses a large, community‐based sample of Medicare Advantage beneficiaries through the SEER‐MHOS linked dataset, allowing for a detailed evaluation of patient‐reported outcomes over an extended follow‐up period. The analysis accounts for a wide range of demographic, clinical, and cancer‐related covariates and confounders. By stratifying patients into mutually exclusive medication groups, this study provides insights into real‐world prescribing patterns and their associations with pain and HRQoL outcomes in long‐term cancer survivors.

### 4.5 Future Direction

This study highlights the persistent burden of pain in cancer survivors and the need for more tailored and multimodal pain management approaches—including non‐pharmacologic approaches (e.g., physical therapy, acupuncture etc.) [30, 32] Future research should focus on identifying which patient subgroups benefit most from gabapentinoids, opioids, or both therapies, particularly in high‐comorbidity populations and also explore how pain type influences treatment effectiveness. Given the lack of significant mental health improvements across groups, studies should also explore integrated approaches addressing both pain and psychological distress.

Further investigation is needed into different therapy's long‐term risks and benefits, including medication adherence, dosing patterns, and side effects. Expanding research into non‐prescription medications, such as the use of over‐the‐counter pain medications, and non‐pharmacologic interventions, such as physical therapy and cognitive‐behavioral therapy, may improve pain management beyond medication use alone. Additionally, larger, more in‐depth analyses of pain medication and extended follow‐up studies are important to understanding long‐term functional outcomes, opioid dependence risks, and quality of life impacts. As healthcare shifts toward opioid‐sparing strategies, further studies will be critical in guiding clinical practice and policy decisions.

## Author Contributions

Shirley K. Chan Sanchez contributed to conceptualization, visualization, drafting of the original manuscript, and critical revision. Samuel V. David contributed to conceptualization, visualization, data curation, formal analysis, drafting of the original manuscript, and critical revision. Efstathia Polychronopoulou, Mukaila Raji, and Yong‐Fang Kuo contributed to conceptualization, supervision, and critical revision of the manuscript, with Yong‐Fang Kuo additionally overseeing project administration. All authors approved the final version of the manuscript.

## Disclosure

*Patient Consent*: The research included de‐identified, publicly available data; therefore, patient consent was not required.

*Plain Language Summary*: Many cancer survivors live with long‐term pain. This study looked at how two types of pain medications, opioids and gabapentinoids, help manage pain and quality of life. We found that gabapentinoids—safer analgesics‐worked about as well as opioids; however, the opioid‐gabapentinoid combination had a modest impact on pain and physical health in a small category of clinically complex survivors with severe pain and multiple chronic conditions. However, pain and mental health still worsened over time for many survivors. The results show the need for safer and effective personalized approaches to pain care that go beyond medications.

## Ethics Statement

The study was approved by the Institutional Review Board (IRB# 24–0196) at the University of Texas Medical Branch.

## Conflicts of Interest

The authors declare no conflicts of interest.

## Supporting information


**Data S1:** cam471189‐sup‐0001‐Tables.docx.

## Data Availability

This study used data from the SEER‐MHOS linked resource. It is available from the National Cancer Institute (NCI) and the Centers for Medicare & Medicaid Services (CMS) with restrictions applied for use. The data was used under license for the current study and are not publicly available. Requests for access can be submitted through https://healthcaredelivery.cancer.gov/seer‐mhos/.
