# Supplementary material for: Comparative Effectiveness of Opioids and Opioid Substitutes on Pain and Health Related Quality of Life Among Cancer Survivors
Source: Cancer Med. 2025 Sep 8;14(17):e71189. doi: 10.1002/cam4.71189 (PMC12415586; doi:10.1002/cam4.71189)
Supplement: Supplementary file 1 — Data S1: cam471189‐sup‐0001‐Tables.docx. [file CAM4-14-e71189-s001.docx]

Supplemental Table-1

Sensitivity Analysis (Excluding those diagnosed before the year 2000)

|  | Change in Pain interference  (fully adjusted model) | | Change in PCS  (fully adjusted model) | | Change in MCS  (fully adjusted model) | |
| --- | --- | --- | --- | --- | --- | --- |
| Characteristics | Estimate (95% CI) | p-value | Estimate (95% CI) | p-value | Estimate (95% CI) | p-value |
| Group |  |  |  |  |  |  |
| Opioid only | REF |  | REF |  | REF |  |
| Gaba only | -0.020 (-0.098,0.058) | 0.609 | -0.163 (-0.875, 0.549) | 0.654 | 0.738 (-0.050, 1.524) | 0.066 |
| Both | -0.106 (-0.177, -0.035) | 0.004 | 0.772 (0.116,  1.428) | 0.021 | -0.507 (-1.200, 0.186) | 0.152 |
| None | -0.005 (-0.044,0.034) | 0.807 | 0.074 (-0.281, 0.429) | 0.681 | 0.236 (-0.139, 0.611) | 0.217 |

Fully adjusted model- adjusted for demographics and cancer characteristics: age at survey, gender, race, smoking category, area, SEER region, education, cancer type, cancer status, comorbidities, stage

**Supplemental Table- 2**

Multivariate analysis for change in pain interference (subgroup analysis for those upto moderate pain, those with quite a bit and extreme pain interfering with work)

|  | Change in pain interference for those upto moderate pain interfering with work  (level 1-3) | | Change in pain interference for those with quite a bit and extreme pain interfering with work  (level 4-5) | |
| --- | --- | --- | --- | --- |
| Characteristics | Estimate (95% CI) | p-value | Estimate (95% CI) | p-value |
| Group |  |  |  |  |
| Opioid only | REF |  | REF |  |
| Gaba only | -0.008(-0.08,0.07) | 0.845 | -0.06(-0.22,0.09) | 0.432 |
| Both | -0.177(-0.25, -0.09) | < 0.0001 | -0.17 (-0.29,-0.05) | 0.0051 |
| None | -0.006(-0.04,0.03) | 0.7534 | -0.09 (-0.008,0.17) | 0.03 |

Fully adjusted model- adjusted for demographics and cancer characteristics- age at survey, gender, race, year group, smoking category, area, SEER region, education, cancer type, cancer status, comorbidities, stage
